# Metabolic impact of feeding prior to a 60-min bout of moderate-intensity exercise in females in a fasted state

**DOI:** 10.3389/fspor.2022.1070477

**Published:** 2023-01-16

**Authors:** Kayla M. Ratliff, Chad M. Kerksick, Jessica M. Moon, Anthony M. Hagele, Johnathan L. Boring, Kylie Walden, Connor J. Gaige, Richard A. Stecker, Kyle L. Sunderland, Petey W. Mumford

**Affiliations:** Exercise and Performance Nutrition Laboratory, Kinesiology Department, College of Science, Technology, and Health, Lindenwood University, St. Charles, MO, United States

**Keywords:** protein, females, fasted exercise, nutrient timing, weight loss, diet

## Abstract

**Background:**

The metabolic impact of pre-exercise feeding of protein or carbohydrate on fat oxidation and energy expenditure rates, especially, in females, is poorly understood.

**Methods:**

Recreationally active females (*n* = 15, 32 ± 10 years, 164.8 ± 5.6 cm, 63.5 ± 9.3 kg, 23.4 ± 3.2 kg/m^2^) completed four testing sessions in a randomized, double-blind, crossover fashion after fasting overnight. Participants ingested isovolumetric and isoenergetic solutions containing either 25 g of whey protein, casein protein, carbohydrate (CHO), or a non-caloric placebo (PLA). Participants then completed 60 min of treadmill exercise at 15% below ventilatory threshold 30 min after ingestion. Respiratory exchange ratio (RER) was evaluated throughout exercise and resting energy expenditure (REE) was assessed pre-exercise, and 0-, 60-, and 120-min post-exercise.

**Results:**

A significant condition x time interaction was observed for RER (*p* = 0.008) during exercise, with CHO exhibiting higher RER values (vs. PLA) at four time points. A significant main effect for condition was observed for carbohydrate (*p* = 0.001) and fat (*p* = 0.02) oxidation rates during exercise, with fat oxidation rates being higher in PLA vs. CHO (*p* = 0.01). When total fat oxidized was calculated across the entire exercise bout, a significant main effect for condition was observed (*p* = 0.01), with PLA being greater than CHO (*p* = 0.04). A significant condition x time interaction (*p* = 0.02) was found for both absolute and normalized REE, with casein and whey protein having significantly higher values than CHO (*p* < 0.05) immediately post-exercise.

**Conclusion:**

When compared to a fasted control (PLA), consuming CHO, but not protein, decreased total fat oxidation prior to a 60-min bout of moderate-intensity exercise in females.

## 1. Introduction

Manipulation of energy stores and feeding approaches prior to exercise continues to be an area of focus for individuals interested in weight loss and augmenting intramuscular adaptations to exercise training (1). For years, the completion of fasted exercise has been postulated as a strategy to increase the utilization of fat during exercise to help people increase fat oxidation and achieve weight loss and physique-oriented goals (2, 3). In concert, feeding during the pre-exercise period has been avoided or, at best, limited due to the potential negative impact feeding may have on resulting fat oxidation rates. Indeed, carbohydrate ingestion increases insulin levels, which effectively blunts fat oxidation (4), but little research has examined the impact of consuming other macronutrients (i.e., protein) during the pre-exercise period on outcomes related to energy expenditure and fat oxidation.

In terms of augmenting energy expenditure and endogenous substrate oxidation, distinct advantages may be present for ingestion of protein. For example, Wingfield et al. (5) has previously reported that pre-exercise ingestion of whey protein (vs. carbohydrate) led to greater rates of energy expenditure and fat oxidation for up to 1 h after exercise was completed. Moreover, Gieske and colleagues (6) used a crossover design in young, healthy men to study the acute impact of isocaloric (25 g) pre-exercise (30-min moderate-intensity treadmill exercise) feedings of whey protein, casein protein, and carbohydrate. In this study, whey or casein protein increased energy expenditure and fat oxidation for up to 60-min post-exercise compared to carbohydrate. Further, Hackney and colleagues (7) have previously reported that pre-exercise ingestion of whey protein increases energy expenditure rates for up to 24 h after ingestion while Paoli et al. (8) concluded that a protein-centric meal before aerobic exercise significantly increases resting metabolism rates for 24 h after exercise. Different types of protein have divergent digestive rates and muscle protein synthesis rates in response to acute feeding (9–11), but research has not fully explored the impact of protein source on substrate oxidation rates during and after acute exercise. Kinsey et al. (12) in men and later by Allman et al. (13) in women have highlighted that ingestion of casein protein prior to sleep does not impact overnight rates of fat oxidation. Alternatively, Hursel and investigators (14) demonstrated that increasing the protein content from 12% to 38% of energy in an early morning meal increases energy expenditure rates.

A key variable that can impact resulting rates of fat oxidation is exercise duration (15, 16). In this respect, Cheneviere et al. (15) noted that fat oxidation continuously increases throughout a 60-min exercise bout, with significantly higher fat oxidation rates after 40 and 60 min of exercise when compared to 20 min. Wingfield et al. (17) and Gieske et al. (6) evaluated the impact of pre-exercise protein feeding on energy expenditure and fat oxidation surrounding exercise bouts that lasted approximately 30 min in duration. As such, any potential augmentation of fat oxidation that may occur during longer (>30 min) bouts of aerobic exercise with pre-exercise protein feeding is largely unknown. In addition, the research completed by Gieske et al. (7) included males while Wingfield et al. (5) included females. Previous research has indicated that gender differences exist in terms of substrate oxidation (18, 19). Towards this end, females have been reported to have higher rates of fat oxidation during exercise, but lower rates of lipolysis, fatty acid metabolism, and total lipid oxidation when compared to men (18, 20). While these gender-specific outcomes are not fully understood, varying levels of estrogen (21) are a primary consideration as changes in estrogen concentration are associated with increased rates of lipolysis, free fatty acid distribution to skeletal muscle, and mRNA expression of many metabolism-related genes (21, 22). The purpose of this study is to test the hypothesis that acute isocaloric feedings of casein or whey protein prior to a 60-min bout of moderate-intensity exercise do not negatively impact rates of fat oxidation throughout and after exercise in comparison to an isocaloric dose of carbohydrate or a non-caloric control in recreationally trained females.

## 2. Methods

### 2.1. Experimental design

**Figure 1:**
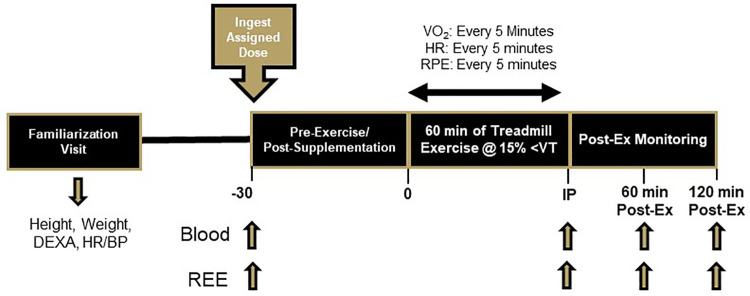
Overview of research design.

This study was a randomized, double-blind, within-subjects, crossover design. Recreationally active, pre-menopausal women that either had regular menstrual cycles or were taking oral contraceptives that resulted in regular cycles were eligible for this study. Participants were screened for eligibility, provided written consent, and completed a health history questionnaire before having resting heart rate, blood pressure, height, weight, and body composition assessed along with their VO_2_Peak on a motorized treadmill. Each participant then completed four separate exercise sessions during the early-follicular phase of their menstrual cycle (within 7 days of the start of menses), with at least 48 h separating visits. Typically, two study visits would be completed within the first 7 days of one cycle, and the last two visits would be completed during the first 7 days of a subsequent cycle. Each exercise session consisted of body weight measurements, pre-exercise blood draw, resting energy expenditure (REE), supplement consumption, and completion of a 60-min treadmill exercise bout at an intensity 15% below their ventilatory threshold (VT). Additional REE measurements were completed 0-, 60-, and 120-min post-exercise (Figure 1). The primary outcomes in this study were observed rates and amounts of fat oxidation.

### 2.2. Study participants

Fifteen healthy, recreationally-active females (32 ± 10 years, 164.8 ± 5.6 cm, 63.5 ± 9.3 kg, 27.1 ± 3.6% fat, 23.4 ± 3.2 kg/m^2^, 36.5 ± 4.2 ml/kg/min) completed the study protocol. All participants were required to abstain from any nutritional supplementation that may alter fat metabolism for at least 30 days prior to the first visit and throughout the duration of the study. All participants reported at least 30 min of moderate-intensity exercise at least 3 days per week, but did not exceed 6 h of aerobic exercise or 8 h of total exercise per week for the previous 6 months. Participants were excluded if they: could not fit on the DEXA table (>136 kg or taller than 198 cm), consumed over 400 mg of caffeine three or more days per week, were using any form of birth control which stopped their menstrual cycle, or were not willing to follow the pre-testing guidelines. The study was approved by the Lindenwood University IRB (IRB-20-175, approval date: 07/27/2020) and all participants provided written prior to any data collection.

### 2.3. Procedures

#### 2.3.1. Anthropometrics and hemodynamics

Participants had their standing height and weight determined using a standardized wall-mounted stadiometer (Tanita, model # HR-200, Tokyo, Japan) and a self-calibrating digital scale (Tanita, model # BWB-627A Class III, Tokyo, Japan). Participants then sat quietly for 3–5 min and resting heart rate and blood pressure was assessed using an automatic blood pressure monitor (OMRON, model # Hem 907XL, Kyoto, Japan).

#### 2.3.2. Body composition

Body composition was determined between 600 and 1,000 h during the familiarization visit *via* dual-energy x-ray absorptiometry (DEXA) (Hologic QDR Discovery A, Bedford, MA). Participants observed an 8–10-h fast from all energy-containing food and drink (including caffeine) and abstained from alcohol, nicotine, tobacco, and exercise for 24 h prior to testing (23). Participants provided a mid-stream urine sample that produced a urine-specific gravity result of ≤1.02 as an indicator of adequate hydration when examined through a hand-held refractometer (General Tools & Instruments, LLC, Secaucus, NJ). DEXA calibration was completed each day and all scans were analyzed using the manufacturer-included software package (Hologic APEX Software, Version 4.5.3) using normative data derived from the 2008 National Health and Nutrition Examination Survey (NHANES) (24).

#### 2.3.3. Dietary records

Participants completed a 2-day food log prior to all testing sessions to assess dietary intake. Food logs were submitted using the National Institutes of Health Automated Self-Administered 24-h dietary recall (ASA24) (25). Each participant was provided with login information and detailed instructions on how to accurately submit the food logs through the ASA24 software. A copy of this food record was then provided to each participant, and they were instructed to replicate their diet for the 2 days prior to each study visit for the remainder of the study.

#### 2.3.4. Supplementation protocol

Subjects were assigned to ingest one of four supplement conditions in a randomized, double-blind, and crossover fashion. A randomized order of treatments was generated by random.org. Prior to study initiation, study supplements were blinded and remained blinded until statistical analysis was completed. The supplement conditions were 25 g of a whey protein isolate (WPI) (Dymatize, Dallas, TX), 25 g of micellar casein (CAS) (Dymatize, Dallas, TX), 25 g of maltodextrin carbohydrate (CHO), or a non-caloric, sweetened control (PLA). Thus, the PLA condition was a fasting control. Outside of PLA, all supplements were isoenergetic and both protein conditions were isonitrogenous. All drinks were similarly colored, flavored, and isovolumetric (eight fluid ounces of cold water mixed with indicated dry powder weights) and were provided to participants in solid non-transparent containers. Participants consumed each drink within 3 min and were then required to sit quietly for 30 min. This dose and timing was chosen to replicate the previous work of Gieske et al. (6) and because previous studies have indicated that muscle protein synthesis rates are stimulated when consuming similar doses at similar times (10).

#### 2.3.5. Resting measures

Expired gas analysis to determine resting energy expenditure rates was completed using a ParvoMedics TrueOne 2400 metabolic measurement system (ParvoMedics, Sandy, UT). After daily calibration of gas and airflow (within 2% of previous day), testing was completed in an isolated, thermoneutral room with the lights illuminated and a clear plastic hood and drape placed over each participant's head and shoulders. Once an appropriate flow rate (0.7%–1.2% CO_2_) was established, study participants remained awake and motionless in a supine position for 20–25 min. The lowest 5-min average was computed for VO_2_ and data for resting energy expenditure was reported in kcals/day.

#### 2.3.6. Peak oxygen consumption

Exercise testing was completed on a Woodway Desmo-Evo treadmill (Woodway USA, Inc., Waukesha, WI USA). During familiarization, participants completed a graded, staged treadmill protocol to assess peak oxygen consumption. The protocol began with a 1-min warm-up walking at 3.0 mph at 1.0% grade. After warm-up completion, the protocol utilized 2-min stages starting at 9.0 km h^−1^ and 1.0% grade and increased in speed by 1.0 km h^−1^ each stage until volitional fatigue was reached. Heart rate was monitored continuously throughout the test (Polar FT1, Polar, Kempele, Finland). A VO_2_Peak was accepted if the respiratory exchange ratio (RER) achieved ≥1.05 and heart rates were within ten beats of the age-predicted maximal heart rate [Max HR = 208—(0.7 × age)]. VO_2_ was averaged over 15-s intervals and VO_2_Peak was reported as the highest VO_2_ value recorded throughout the exercise test. VT was determined as the point where the ventilatory equivalents of oxygen (Ve/VO_2_) and carbon dioxide (Ve/VCO_2_) intersected.

#### 2.3.7. Exercise bouts

The average time between visits during the same menstrual cycle was approximately 4 days (2 cycles, *n* = 8; 3 cycles, *n* = 6; 4 cycles, *n* = 1). Thirty minutes after ingestion of their assigned supplement, each participant completed separate 60-min bouts of treadmill exercise using a speed and grade combination which yielded exercise VO_2_ values that were approximately 15% below their individual VT. To account for differences in comfort and efficiency of walking, treadmill speed for all participants was fixed at 3.0 mph and grade was predicted using the American College of Sports Medicine (ACSM) walking equation. Using this approach, exercise VO_2_ ranged between 56% and 76% VO_2_Peak. After calculations were completed, no changes in speed or grade were made and expired gas kinetics were assessed using indirect calorimetry every 5 min during the exercise bout. Heart rate was assessed every 5 min using a Polar FT1 heart rate transmitter worn on the chest.

Substrate oxidation rates were calculated according to the methods of Frayn (26) and total fat oxidation during each 5-min period was calculated using standard thermal equivalents of oxygen using the non-protein respiratory quotient (NPRQ) table (17, 27).

#### 2.3.8. Blood collection and analysis

Venous blood samples were collected 30 min pre-exercise during each study visit and centrifuged at room temperature for 10 min at 3,500 × *g*. All tubes were stored at −80°C for later analysis of estradiol using a commercial microplate assay from DRG International (Springfield, NJ) at a wavelength of 450 nm.

### 2.4. Statistical analysis

All data is presented as means ± standard deviations and analyzed using IBM SPSS 26 (Armonk, NY USA) and graphs were generated using GraphPad (La Jolla, CA). Energy expenditure data was normalized to body weight in kilograms. Mixed (condition × time) factorial ANOVAs were used to assess differences between conditions over time. Furthermore, if significant interactions were found then data were further decomposed by repeated measures ANOVA across time within each condition or across conditions at each time point, and *post-hoc* testing was conducted with Bonferroni correction factors applied. If the assumption of heteroscedasticity for repeated measures were violated, a Greenhouse-Geisser correction factor was applied. Statistical significance for all null hypothesis testing was set at *p *≤ 0.05. No *a priori* power analysis was completed. Sample size was established based upon our previous work in healthy males using a similar study design (6).

## 3. Results

### 3.1. Demographics

Supplementary Data File S1 outlines mean data values for all participants (*n* = 15). Body weight did not change (*p* = 0.30) across the study protocol. Additionally, baseline estrogen levels were not different across conditions (*p* = 0.37) (CHO: 61.0 ± 44.4 pg/ml; Casein: 66.0 ± 72.0 pg/ml; Whey: 59.8 ± 30.5 pg/ml; PLA: 53.8 ± 23.6 pg/ml).

### 3.2. Dietary intake

No significant differences (*p* > 0.05) were observed between conditions for total energy intake (kcal/day) or absolute (g/day) and relative (g/kg/day) carbohydrate, fat, and protein consumption (Supplementary Data File S1).

### 3.3. Exercise intensity

No significant condition x time interactions were observed for heart rate (*p* = 0.66), VO_2_ (*p* = 0.52), or energy utilization (*p* = 0.56) (Supplementary Data File S2). However, significant main effects of time were observed for heart rate (*p* < 0.001) and VO_2_ (*p* < 0.001) and a main effect for condition for VO_2_ (*p* = 0.001). Heart rate and VO_2_ values increased throughout exercise for all conditions. Exercise VO_2_ values across the entire exercise bout were greater in casein than PLA and CHO and greater in whey than PLA (Supplementary Data File S2).

### 3.4. Intra-Exercise substrate oxidation

A significant condition x time interaction was observed for respiratory exchange ratio (*p* = 0.008). Respiratory exchange ratio was significantly greater in CHO vs. PLA (*p* < 0.05) at the 10–15-, 20–25-, 30–35-, and 40–45-min time points throughout exercise. In addition, casein was higher than CHO (*p* < 0.05) at the 30–35-min time point.

**Figure 2:**
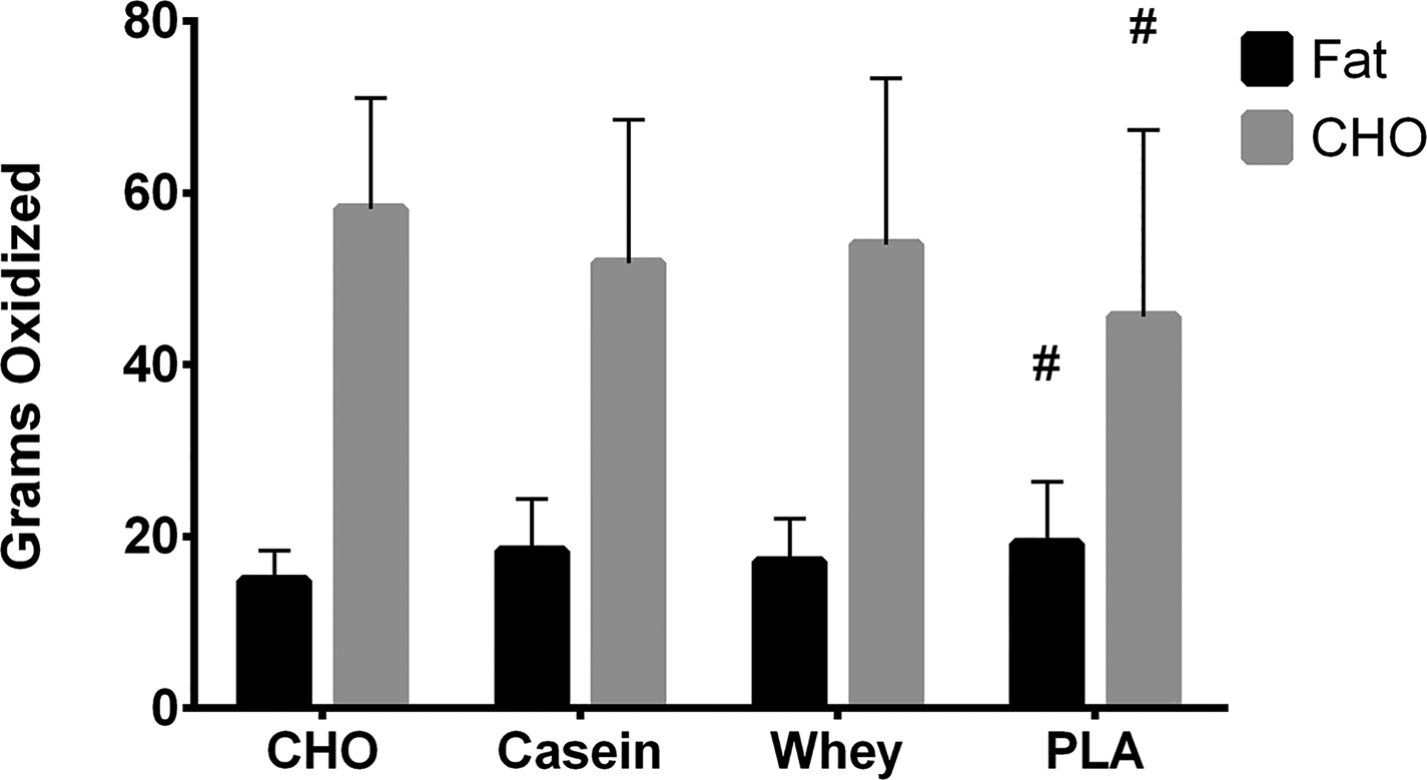
Total grams of fat and carbohydrate oxidized over the 60-min exercise bout for each condition. #, different than CHO (*p* < 0.05).

No significant condition x time interactions were observed for carbohydrate oxidation rate (*p* = 0.12) or fat oxidation rate (*p* = 0.20) (Supplementary Data File S3). However, significant main effects were observed for carbohydrate (time, *p* < 0.001 and condition, *p* = 0.001) and fat oxidation rates (time, *p* < 0.001 and condition, *p* = 0.021). Carbohydrate oxidation rates decreased throughout exercise for all conditions, and total carbohydrate oxidized was lower in PLA (46.3 ± 20.7 g) vs. CHO (57.0 ± 12.9 g) (Supplementary Data File S3). Fat oxidation rates increased throughout exercise for all conditions, and total fat oxidation was higher (*p* = 0.04) in PLA (19.4 ± 7.4 g) vs. CHO (15.1 ± 3.5 g) (Figure 2).

A significant main effect for condition was observed for total energy expended throughout the exercise bout (*p* = 0.010). Casein (389 ± 57 kcals) and whey (385 ± 51 kcals) had significantly higher total energy expenditure when compared to PLA (PLA: 370 ± 56 kcals).

### 3.5. Resting energy expenditure

#### 3.5.1. Absolute resting energy expenditure

A significant condition x time interaction was observed for absolute resting energy expenditure (*p* = 0.02) (Supplementary Data File S4). Significant main effects were observed for time (*p* < 0.001) and condition (*p* = 0.001). At the immediate post-exercise time point, whey (1,603 ± 161 kcals/day) and casein (1,657 ± 270 kcals/day) both had significantly greater values than CHO (1,454 ± 173 kcals/day) and PLA (1,503 ± 212 kcal/day).

#### 3.5.2. Normalized resting energy expenditure

**Figure 3:**
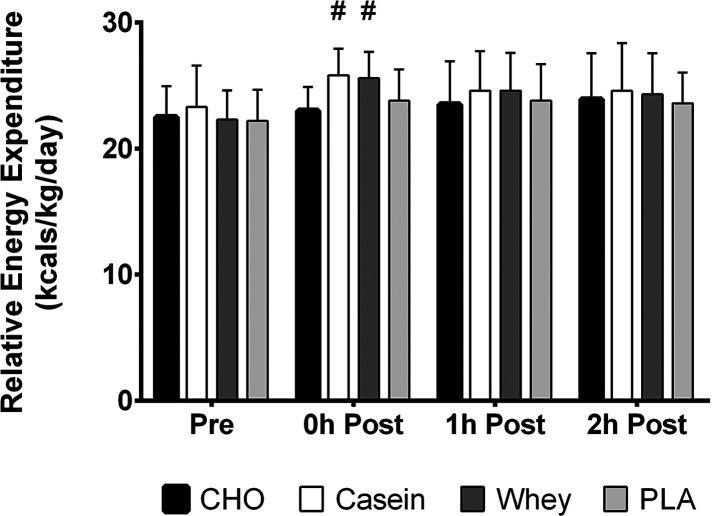
REE relative to body weight in kilograms over all four time points in each condition. #, different than CHO (*p* < 0.05).

A significant condition x time interaction was observed for normalized resting energy expenditure values (*p* = 0.02). Significant main effects were observed for time (*p* < 0.001) and condition (*p* = 0.005). Simple main effects analysis revealed casein and whey had significantly greater values than CHO at the immediate post-exercise time point (Figure 3). No other differences in normalized REE were observed.

### 3.6. Respiratory exchange ratio

No significant condition x time interaction (*p* = 0.83) or main effect of condition (*p* = 0.80) was observed for respiratory exchange ratio. However, a significant main effect of time was observed (*p* = 0.007), showing a decrease in REE from pre- to post-exercise.

## 4. Discussion

This study examined, against a fasted condition, the changes in substrate oxidation and energy expenditure in healthy females after consuming isocaloric doses of whey protein, casein protein, or carbohydrate 30 min prior to a 60-min bout of moderate-intensity (56%–76% VO_2_Peak) treadmill exercise. The findings from this study indicate that pre-exercise protein feeding, when compared to fasted exercise, did not significantly impact carbohydrate or fat oxidation during exercise. Alternatively, pre-exercise CHO feeding significantly increased total carbohydrate oxidation and decreased total fat oxidation when compared to fasted exercise. In addition, energy expenditure rates immediately after exercise were greater with protein ingestion when compared to CHO.

Results from the current study indicate that, in women, fasting prior to a moderate-intensity 60-min exercise bout results in significantly greater total fat oxidation compared to pre-exercise carbohydrate consumption. These findings are discordant with Gieske et al. (6) who reported, in healthy men, that isonitrogenous doses of whey or casein protein 30 min prior to a 30-min bout of moderate-intensity (55%–60% heart rate reserve) treadmill exercise resulted in significantly higher rates of fat oxidation post-exercise compared to CHO or fasting. A key difference between the studies is that Gieske et al. (6) examined male subjects and previous research has shown that women oxidize a higher proportion of lipids when compared to men throughout several exercise mode and intensity combinations (18, 19, 28). Previous work by Wingfield et al. (5) in younger women reported that whey protein led to lower respiratory exchange ratio values after acute bouts of exercise when compared to performing the same exercise while fasted. While our work failed to show superiority of either protein source, it did demonstrate that protein ingestion did not negatively impact respiratory exchange ratio values in comparison to exercising in a fasted state. The present study extended the findings of Gieske et al. to include follow-up metabolic measurements 60 and 120-min post-exercise. In the present study, absolute REE (kcals/day) significantly increased at all post-exercise time points in whey, while casein ingestion and the fasting condition resulted in absolute energy expenditure increases immediately and 60-min post-exercise. When normalized to body mass, casein and whey protein resulted in significantly greater energy expenditure levels immediately after exercise when compared to CHO. While post-exercise energy expenditure rates differed, no differences between conditions were observed found for RER values measured during the post-exercise measurements. Previously, Horton et al. (29) observed that the contribution of fat towards energy increased significantly in both men and women during post-exercise recovery after a 120-min bout of cycling at 40% VO_2_Max. However, exercise in this study was longer and was performed at a lower intensity (40% VO_2_max) when compared to the present study, which may have resulted in a higher contribution of lipids being used for energy (30). Collectively, these outcomes suggest that in healthy, recreational women, pre-exercise ingestion of either whey or casein protein ingestion does not disrupt substrate oxidation rates when compared to exercising in a fasted state or when consuming an isocaloric dose of carbohydrate. Additionally, consuming carbohydrate prior to exercise increases carbohydrate oxidation and blunts fat oxidation when compared to fasted exercise (i.e., our PLA condition).

Previous studies have shown that women exhibit higher values of lipid oxidation during the luteal phases of the menstrual cycle compared to follicular (31, 32). For this reason, care was taken to control for the impact of varying hormonal levels and all participants were tested in the early follicular phase of their menstrual cycle (5–7 days after the start of menstruation). Notably, serum estrogen levels were similar prior to each condition (CHO: 61.0 ± 44.4 pg/ml; Casein: 66.0 ± 72.0 pg/ml; Whey: 59.8 ± 30.5 pg/ml; PLA: 53.8 ± 23.6 pg/ml, *p* = 0.37). Eumenorrheic (*n* = 10) and women regulating their cycle with oral contraceptives (*n* = 5) were included in the study as previous research by Isacco et al. (33) has indicated that oral contraceptives exert little impact on substrate metabolism during a cycling exercise bout at 65% VO_2_Max. Further research is needed to see if these types of birth control would have significant effects on substrate oxidation during exercise.

Throughout each exercise bout, collected VO_2_ values were shown to be significantly different between conditions while no differences between conditions were observed for heart rate or energy utilization (Supplementary Data File S2). The greater VO_2_ values observed for casein and whey was somewhat unexpected as the order of treatment administration was randomized throughout the study protocol. Previous research by Wiles et al. (34) did report higher exercise VO_2_ when a 0.4 g/kg dose of whey isolate was ingested 60 min before exercise when compared to ingesting the same dose 180 min prior to exercise. The results from the Wiles study and the present study are likely due to the higher thermic effect of food that is associated with protein consumption or digestion in general (35). It is likely these greater exercise VO_2_ rates could have explained our greater post-exercise energy expenditure rates immediately post-exercise, but the impact of these differences on our observed substrate oxidation rates is challenging to highlight. One could view that pre-exercise ingestion of protein (irrespective of the source) leads to a reduction in exercise economy, but our study was not appropriately designed to evaluate differences in exercise economy.

Limitations to our study include the lack of protein oxidation measurement in addition to assessment of biomarkers that could provide deeper insight into carbohydrate and fat oxidation. The formulas used to calculate carbohydrate and fat oxidation assume that rates of protein oxidation are negligible (5%–10%) and changes only marginally across similar exercise bouts. As such, we cannot rule out that changes in protein oxidation rates after protein ingestion impacted our observed outcomes, however, we can say that protein ingestion did yield similar rates of fat oxidation as fasted exercise. Thus, for those women who want to take advantage of the benefits of protein ingestion without negatively impacting how much fat is oxidized during their exercise bouts, this may be meaningful. Additionally, this study expanded on the post-exercise measurements completed by Gieske et al. (6) by adding post-exercise measurements up to 120-min post exercise and whey still had absolute REE values that were significantly higher than baseline at the 120-min post-exercise timepoint. As such, post-exercise energy utilization rates were still increasing suggesting that post-exercise time points may need to be extended further. To control for the impact of circulating estrogen on substrate oxidation, we commonly scheduled two visits within 72 h of each other during the first 7 days of the menstrual cycle. Therefore, visits this close together may have impacted our measurements during a subsequent visit, but McLester and others (36) have indicated that recovery periods of at least 48 h allow for adequate recovery. Regardless, we feel the strength of controlling for circulating endogenous hormone levels throughout all visits superseded any potential limitations this approach created.

## 5. Conclusion

In healthy females, carbohydrate consumption prior to a 60-min bout of moderate-intensity treadmill exercise decreases total fat oxidation when compared to a fasted placebo group while no differences in fat oxidation were observed between fasting and protein consumption. Energy expenditure rates after exercise and protein ingestion significantly increased over pre-exercise REE rates and were significantly greater than CHO immediately after exercise. Future research should examine these changes in responses to resistance exercise, a combination of aerobic and resistance exercise, or some patterns of high-intensity intervals. Additionally, a longer period of post-exercise resting metabolic measurements might be needed to fully assess the magnitude of post-exercise increases in energy expenditure in combination with pre-exercise feeding.

## Data Availability

The raw data supporting the conclusions of this article will be made available by the authors upon reasonable request.
