# Supplementary material for: Metabolic impact of feeding prior to a 60-min bout of moderate-intensity exercise in females in a fasted state
Source: Front Sports Act Living. 2023 Jan 16;4:1070477. doi: 10.3389/fspor.2022.1070477 (PMC9884971; doi:10.3389/fspor.2022.1070477)
Supplement: Supplementary file 1 [file Datasheet1.docx]

**Supplementary Data File 1.** Selected dietary variables.

| Variable | CHO | Casein | Whey | PLA | (*p*) |
| --- | --- | --- | --- | --- | --- |
| Energy Intake (kcal/day) | 1740 ± 579 | 1824 ± 804 | 1569 ± 751 | 1633 ± 656 | 0.69 |
| Relative Energy Intake (kcal/kg) | 27.3 ± 8.2 | 28.1 ± 12.7 | 25.3 ± 12.2 | 25.9 ± 10.7 | 0.85 |
| Carbohydrate (g/day) | 165.2 ± 60.6 | 174.6 ± 91.5 | 157.2 ± 95.5 | 161.7 ± 70.9 | 0.83 |
| Relative Carbohydrate (g/kg/day) | 2.6 ± 0.9 | 2.7 ± 1.4 | 2.5 ± 1.5 | 2.6 ± 1.1 | 0.97 |
| Protein (g/day) | 105.1 ± 44.1 | 91.4 ± 42.9 | 84.8 ± 41.7 | 90.1 ± 38.8 | 0.47 |
| Relative Protein (g/kg) | 1.6 ± 0.6 | 1.4 ± 0.7 | 1.4 ± 0.7 | 1.4 ± 0.6 | 0.57 |
| Fat (g/day) | 71.5 ± 27.0 | 76.4 ± 41.6 | 66.0 ± 37.7 | 66.3 ± 33.2 | 0.80 |
| Relative Fat (g/kg) | 1.1 ± 0.4 | 1.2 ± 0.7 | 1.1 ± 0.6 | 1.1 ± 0.5 | 0.84 |

All data presented as Mean ± SD.
