# Supplementary material for: Metabolic impact of feeding prior to a 60-min bout of moderate-intensity exercise in females in a fasted state
Source: Front Sports Act Living. 2023 Jan 16;4:1070477. doi: 10.3389/fspor.2022.1070477 (PMC9884971; doi:10.3389/fspor.2022.1070477)
Supplement: Supplementary file 2 [file Datasheet2.docx]

**Supplementary Data File 2.** Exercise heart rate, VO_2_, and energy expended throughout exercise bout.

| Variables | Time | CHO | Casein | Whey | PLA | p-value | |
| --- | --- | --- | --- | --- | --- | --- | --- |
| Heart Rate  (beats/min) | 0-5 | 120 ± 23 | 129 ± 24 | 130 ± 19 | 113 ± 45 | Condition | 0.53 |
|  | 10-15 | 133 ± 20 | 135 ± 25 | 136 ± 21 | 135 ± 21 | Time | <0.001 |
|  | 20-25 | 134 ± 21 | 138 ± 25 | 138 ± 23 | 136 ± 20 | C x T | 0.66 |
|  | 30-35 | 136 ± 21 | 138 ± 26 | 139 ± 23 | 139 ± 22 |  |  |
|  | 40-45 | 136 ± 21 | 138 ± 26 | 139 ± 23 | 140 ± 21 |  |  |
|  | 50-55 | 136 ± 20 | 139 ± 26 | 141 ± 23 | 142 ± 20 |  |  |
| VO_2_  (L/min) | 0-5 | 20.1 ± 1.3 | 20.5 ± 1.9 | 20.7 ± 1.8 | 19.3 ± 2.3 | Condition | 0.001 |
|  | 10-15 | 20.1 ± 1.3 | 20.8 ± 1.4 | 20.7 ± 1.6 | 20.2 ± 1.9 | Time | <0.001 |
|  | 20-25 | 20.2 ± 1.4 | 21.0 ± 1.4 | 20.5 ± 1.6 | 20.3 ± 1.8 | C x T | 0.52 |
|  | 30-35 | 20.6 ± 1.3 | 21.1 ± 1.5 | 21.2 ± 1.6 | 20.4 ± 1.7 |  |  |
|  | 40-45 | 20.5 ± 1.6 | 21.3 ± 1.4 | 21.2 ± 1.6 | 20.1 ± 1.9 |  |  |
|  | 50-55 | 20.8 ± 1.5 | 21.5 ± 1.4 | 21.2 ± 1.6 | 20.5 ± 1.8 |  |  |
| Kcals/min | 0-5 | 6.1 ± 0.8 | 6.2 ± 1.0 | 6.2 ± 0.8 | 5.9 ± 1.0 | Condition | 0.23 |
|  | 10-15 | 6.1 ± 0.7 | 6.3 ± 0.9 | 6.2 ± 0.9 | 6.1 ± 0.8 | Time | 0.001 |
|  | 20-25 | 6.2 ± 0.8 | 6.4 ± 0.9 | 6.3 ± 0.8 | 5.7 ± 1.8 | C x T | 0.085 |
|  | 30-35 | 6.3 ± 0.8 | 6.4 ± 0.9† | 6.3 ± 0.7 | 6.1 ± 0.8 |  |  |
|  | 40-45 | 6.3 ± 0.8 | 6.4 ± 0.94 | 6.2 ± 0.7 | 6.1 ± 0.9 |  |  |
|  | 50-55 | 6.2 ± 0.8 | 6.5 ± 0.9 | 6.3 ± 0.6 | 6.2 ± 0.8 |  |  |

C x T = Condition x Time.
