# Supplementary material for: Metabolic impact of feeding prior to a 60-min bout of moderate-intensity exercise in females in a fasted state
Source: Front Sports Act Living. 2023 Jan 16;4:1070477. doi: 10.3389/fspor.2022.1070477 (PMC9884971; doi:10.3389/fspor.2022.1070477)
Supplement: Supplementary file 3 [file Datasheet3.docx]

**Supplementary Data File 3.** Intra-exercise respiratory exchange ratio, rates of carbohydrate and fat oxidation, and total fat and carbohydrate oxidation.

| Variables | Time | PLA | CHO | Casein | Whey | p-value | |
| --- | --- | --- | --- | --- | --- | --- | --- |
| Respiratory Exchange Ratio | 0-5 | 0.85 ± 0.06 | 0.88 ± 0.04 | 0.86 ± 0.04 | 0.87 ± 0.06 | Condition | 0.004 |
|  | 10-15 | 0.86 ± 0.05# | 0.89 ± 0.02 | 0.87 ± 0.04 | 0.87 ± 0.04 | Time | <0.001 |
|  | 20-25 | 0.84 ± 0.06# | 0.89 ± 0.02 | 0.86 ± 0.04 | 0.86 ± 0.04 | C x T | 0.008 |
|  | 30-35 | 0.83 ± 0.06# | 0.88 ± 0.03 | 0.85 ± 0.04# | 0.85 ± 0.05 |  |  |
|  | 40-45 | 0.82 ± 0.05# | 0.86 ± 0.03 | 0.84 ± 0.04 | 0.85 ± 0.04 |  |  |
|  | 50-55 | 0.82 ± 0.05 | 0.85 ± 0.03 | 0.84 ± 0.04 | 0.83 ± 0.05 |  |  |
| Carbohydrate Oxidation Rate  (g/min) | 0-5 | 0.74 ± 0.33 | 0.96 ± 0.26 | 0.85 ± 0.29 | 0.90 ± 0.35 | Condition | 0.001 |
|  | 10-15 | 0.82 ± 0.29 | 1.03 ± 0.14 | 0.91 ± 0.22 | 0.94 ± 0.29 | Time | <0.001 |
|  | 20-25 | 0.72 ± 0.34 | 1.01 ± 0.17 | 0.88 ± 0.23 | 0.90 ± 0.32 | C x T | 0.115 |
|  | 30-35 | 0.68 ± 0.32 | 1.00 ± 0.21 | 0.83 ± 0.24 | 0.83 ± 0.31 |  |  |
|  | 40-45 | 0.62 ± 0.33 | 0.87 ± 0.18 | 0.79 ± 0.24 | 0.79 ± 0.27 |  |  |
|  | 50-55 | 0.62 ± 0.28 | 0.80 ± 0.22 | 0.76 ± 0.22 | 0.73 ± 0.29 |  |  |
| Fat Oxidation Rate  (g/min) | 0-5 | 0.29 ± 0.13 | 0.23 ± 0.08 | 0.28 ± 0.09 | 0.27 ± 0.11 | Condition | 0.015 |
|  | 10-15 | 0.29 ± 0.12 | 0.20 ± 0.05 | 0.27 ± 0.09 | 0.25 ± 0.07 | Time | <0.001 |
|  | 20-25 | 0.32 ± 0.13 | 0.22 ± 0.07 | 0.28 ± 0.09 | 0.28 ± 0.09 | C x T | 0.198 |
|  | 30-35 | 0.34 ± 0.13 | 0.22 ± 0.06 | 0.30 ± 0.10 | 0.30 ± 0.10 |  |  |
|  | 40-45 | 0.36 ± 0.13 | 0.28 ± 0.08 | 0.33 ± 0.11 | 0.31 ± 0.10 |  |  |
|  | 50-55 | 0.37 ± 0.12 | 0.32 ± 0.13 | 0.34 ± 0.11 | 0.34 ± 0.10 |  |  |
| Total fat oxidized (grams) | | 19.4 ± 7.4# | 15.1 ± 3.5 | 18.1 ± 6.2 | 16.4 ± 4.6 | *p* | 0.011 |
| Total carbohydrate oxidized (grams) | | 46.3 ± 20.7# | 57.0 ± 12.9 | 51.5 ± 16.6 | 50.3 ± 19.6 | *p* | 0.002 |

C x T = Condition x Time; # = Different than CHO (p<0.05).
