# Supplementary material for: Metabolic impact of feeding prior to a 60-min bout of moderate-intensity exercise in females in a fasted state
Source: Front Sports Act Living. 2023 Jan 16;4:1070477. doi: 10.3389/fspor.2022.1070477 (PMC9884971; doi:10.3389/fspor.2022.1070477)
Supplement: Supplementary file 4 [file Datasheet4.docx]

**Supplementary Data File 4.** Post-exercise resting energy expenditure and respiratory exchange ratio data

|  | Time | PLA | CHO | Casein | Whey | p-value | |
| --- | --- | --- | --- | --- | --- | --- | --- |
| Absolute REE (kcal/day) | Pre-Ex | 1396 ± 182 | 1414 ± 143 | 1483 ± 228 | 1391 ± 139 | Condition | 0.001 |
|  | Immed Post | 1503 ± 212 | 1454 ± 173 | 1657 ± 270#§* | 1603 ± 161#§* | Time | <0.001 |
|  | 60 min Post | 1500 ± 185* | 1478 ± 232 | 1569 ± 230* | 1534 ± 184* | C x T | 0.02 |
|  | 120 min Post | 1486 ± 179* | 1495 ± 197 | 1558 ± 196 | 1526 ± 248* |  |  |
| Relative REE (kcal/kg/day) | Pre-Ex | 22.2 ± 2.5 | 22.5 ± 2.6 | 23.3 ± 3.3 | 22.3 ± 2.3 | Condition | 0.005 |
|  | Immed Post | 23.8 ± 2.5 | 23.0 ± 1.9 | 25.8 ± 2.1†* | 25.6 ± 2.1†* | Time | <0.001 |
|  | 60 min Post | 23.9 ± 2.9 | 23.5 ± 3.4 | 24.6 ± 3.1* | 24.6 ± 3.0* | C x T | 0.02 |
|  | 120 min Post | 23.6 ± 2.5* | 23.9 ± 3.7 | 24.6 ± 3.8 | 24.3 ± 3.3* |  |  |
| Respiratory Exchange Ratio | Pre-Ex | 0.81 ± 0.07 | 0.80 ± 0.06 | 0.78 ± 0.08 | 0.80 ± 0.08 | Condition | 0.80 |
|  | Immed Post | 0.77 ± 0.05 | 0.75 ± 0.05 | 0.75 ± 0.07 | 0.77 ± 0.07 | Time | 0.007 |
|  | 60 min Post | 0.78 ± 0.06 | 0.77 ± 0.05 | 0.77 ± 0.06 | 0.78 ± 0.05 | C x T | 0.83 |
|  | 120 min Post | 0.79 ± 0.05 | 0.78 ± 0.04 | 0.78 ± 0.06 | 0.78 ± 0.06 |  |  |

C x T = Condition x Time; REE = Resting energy expenditure. # = Different than CHO (p<0.05). § Different than PLA (p<0.05). * = Different than pre-ex (p<0.05).
